# Does breast carcinoma belong to the Lynch syndrome tumor spectrum? – Somatic mutational profiles vs. ovarian and colorectal carcinomas

**DOI:** 10.18632/oncotarget.27538

**Published:** 2020-04-07

**Authors:** Noora K. Porkka, Alisa Olkinuora, Teijo Kuopio, Maarit Ahtiainen, Samuli Eldfors, Henrikki Almusa, Jukka-Pekka Mecklin, Päivi Peltomäki

**Affiliations:** ^1^Department of Medical and Clinical Genetics, University of Helsinki, Helsinki, Finland; ^2^Department of Pathology, Jyväskylä Central Hospital, Jyväskylä, Finland; ^3^Department of Biological and Environmental Science, University of Jyväskylä, Jyväskylä, Finland; ^4^Department of Education and Research, Jyväskylä Central Hospital and University of Eastern Finland, Jyväskylä, Finland; ^5^Institute for Molecular Medicine Finland, University of Helsinki, Helsinki, Finland; ^6^Faculty of Sport and Health Sciences, University of Jyväskylä, Jyväskylä, Finland; ^7^Department of Surgery, Jyväskylä Central Hospital, Jyväskylä, Finland; ^8^Department of Education & Science, Jyväskylä Central Hospital, Jyväskylä, Finland

**Keywords:** Lynch syndrome, breast carcinoma, MSI, DNA mismatch repair, somatic mutation

## Abstract

Inherited DNA mismatch repair (MMR) defects cause predisposition to colorectal, endometrial, ovarian, and other cancers occurring in Lynch syndrome (LS). It is unsettled whether breast carcinoma belongs to the LS tumor spectrum. We approached this question through somatic mutational analysis of breast carcinomas from LS families, using established LS-spectrum tumors for comparison. Somatic mutational profiles of 578 cancer-relevant genes were determined for LS-breast cancer (LS-BC, *n* = 20), non-carrier breast cancer (NC-BC, *n* = 10), LS-ovarian cancer (LS-OC, *n* = 16), and LS-colorectal cancer (LS-CRC, *n* = 18) from the National LS Registry of Finland. Microsatellite and MMR protein analysis stratified LS-BCs into MMR-deficient (dMMR, *n* = 11) and MMR-proficient (pMMR, *n* = 9) subgroups. All NC-BCs were pMMR and all LS-OCs and LS-CRCs dMMR. All but one dMMR LS-BCs were hypermutated (> 10 non-synonymous mutations/Mb; average 174/Mb per tumor) and the frequency of MMR-deficiency-associated signatures 6, 20, and 26 was comparable to that in LS-OC and LS-CRC. LS-BCs that were pMMR resembled NC-BCs with respect to somatic mutational loads (4/9, 44%, hypermutated with average mutation count 33/Mb vs. 3/10, 30%, hypermutated with average 88 mutations/Mb), whereas mutational signatures shared features of dMMR LS-BC, LS-OC, and LS-CRC. Epigenetic regulatory genes were significantly enriched as mutational targets in LS-BC, LS-OC, and LS-CRC. Many top mutant genes of our LS-BCs have previously been identified as drivers of unselected breast carcinomas. In conclusion, somatic mutational signatures suggest that conventional MMR status of tumor tissues is likely to underestimate the significance of the predisposing MMR defects as contributors to breast tumorigenesis in LS.

## INTRODUCTION

Lynch syndrome (LS) is a prevalent cancer predisposition syndrome, originally defined by the Amsterdam criteria [1, 2] and later by pathogenic or likely pathogenic germline variants of the DNA mismatch repair (MMR) genes *MLH1*, *MSH2*, *MSH6*, or *PMS2* [3]. Compared to the general population, carriers of such variants have significantly increased risks of cancers of the colon and rectum, endometrium, ovary, kidney and urinary tract, upper gastrointestinal tract, and certain other organs [4]. In comparison with earlier retrospective and family-based studies, recent prospective studies have arrived at somewhat lower age-specific risk estimates for cancers occurring in MMR variant carriers; moreover, penetrance and expression patterns greatly depend on the MMR gene involved [5]. Among individual MMR genes, pathogenic variants in *MLH1* and *MSH2* have the highest and *PMS2* the lowest penetrance, and *MSH6* variants underlie a sex-limited trait with a high risk of gynecological cancers in females [5].

Evaluations of breast cancer risk in LS have arrived at conflicting findings. Win et al. [6] conducted a systematic review on breast cancer in LS and identified 8 studies reporting an elevated (2–18-fold) risk, whereas the remaining 13 studies found no significantly increased risk. Moreover, breast cancer risk has been reported to be specifically associated with certain MMR genes, including *MLH1* (vs. *MSH2* [7, 8]), *MSH2* [9], and *MSH6* and *PMS2* (vs. *MLH1* and *MSH2* [10, 11]. Variability in results may reflect different methods of ascertainment, cohort sizes, ethnicity, or other factors. In the large multicenter prospective investigation by Dominguez-Valentin et al. [5], the cumulative risk of breast cancer to 75 years of age was 12–15%, similar across all four MMR genes and representing only a marginal increase vs. average population.

Instability at microsatellite sequences (MSI) and absent MMR protein expression by immunohistochemical analysis of tumor tissues are common pre-screening methods for LS. Such methods classified 51% (62/122) of breast carcinomas from predisposing MMR gene variant carriers as MMR-deficient (dMMR) in studies reviewed by Win et al. [6]. Since deficient MMR is rare (< 2%) in breast carcinomas from the average population [12], the result implied a role for MMR deficiency in LS-associated breast cancer. Furthermore, abnormal immunohistochemistry and hypermutated tumor phenotype, combined with early onset of the disease (29 years), recently led to the suggestion that breast cancer is part of the tumor spectrum of the constitutional mismatch repair deficiency (CMMRD) syndrome caused by biallelic pathogenic germline variants of MMR genes [13].

We previously showed that breast carcinoma from carriers of inherited MMR defects resembles common breast carcinoma with respect to many clinicopathological features, such as mean age at onset over 50 years; however, the fact that over half of the tumors were dMMR suggested etiologic association to LS [14]. In the present investigation, we use somatic mutation profiling of breast cancers vs. established LS-spectrum tumors as a tool to address the relationship between breast cancer and LS.

## RESULTS

### Clinicopathological characteristics of patients and tumors

This study was designed to investigate if breast carcinoma, the most common form of cancer in the general female population, is in LS individuals molecularly associated with their inherited MMR defects. To this end, carcinomas of the breast (BC), ovary (OC), and colon and rectum (CRC) were ascertained through the National LS Registry of Finland. Breast carcinomas from carriers of pathogenic or likely pathogenic germline MMR variants (LS-BC, *n* = 20) were compared to breast carcinomas from patients shown not to carry the predisposing MMR gene variant of their families (NC-BC, *n* = 10) and to established LS-spectrum tumors from pathogenic MMR gene variant carriers (LS-OC, *n* = 16, and LS-CRC, *n* = 18) (Tables 1 and 2). The different predisposing MMR genes were roughly similarly distributed across all LS groups, with *MLH1* associated with 67%, *MSH2* with 17%, and *MSH6* with 17% of the total 54 tumors (Table 2).

**Table 1:** Molecular characteristics of breast carcinomas case by case

	Sample ID	Predisposing germline variant	MMR protein IHC	MSI status	Second hit status^*^	Mutations/Mb
**dMMR LS-BC**	BC5_31814T	*MLH1* ex 16, 3.5 kb genomic deletion (Mut I)	negat	MSS	L	141
	BC6_31489T	*MLH1* ex 16, 3.5 kb genomic deletion (Mut I)	negat	MSI	L, s	725
	BC11_31501T	*MLH1* ex 16, 3.5 KB genomic deletion (Mut I)	negat	MSI	L	24
	BC11_31533T	*MLH1* ex 16, 3.5 Kb genomic deletion (Mut I)	negat	MSS	L	5
	BC12_31491T	*MLH1* ex 16, 3,5 kb genomic deletion (Mut I)	negat	MSI	None	22
	BC14_33229T	*MLH1* ex 16, 3.5 kb genomic deletion (Mut I)	negat	MSS	s	662
	BC9_33225T	*MLH1* c.454-1G>A (mutation II)	negat	MSI	L	29
	BC7_33223T	*MSH2* c.187delG	negat	MSI	L	15
	BC37_33228T	*MSH2* ex 1-7 genomic deletion/MLPA	negat	MSI	s	12
	BC1_33203T	*MSH6* ex 1-2 genomic deletion/MLPA	negat	MSS	s	35
	BC1_31528T	*MSH6* ex 1-2 genomic deletion/MLPA	negat	MSS	s	246
**pMMR LS-BC**	BC5_31495T	*MLH1* ex 16, 3.5 kb genomic deletion (Mut I)	posit	MSS	None	31
	BC13_31486T	*MLH1* ex 16, 3.5 kb genomic deletion (Mut I)	posit	MSS	s	85
	BC4_31514T	*MLH1* ex 16, 3.5 kb genomic deletion (Mut I)	posit	MSS	L	4
	BC38_31510T	*MLH1* c.454-1G>A (mutation II)	posit	MSS	s	53
	BC8_33226T	*MSH2* ex 1-16 genomic deletion/MLPA	posit	MSS	None	8
	BC10_33221T	*MSH2 c.*1738insA	posit	MSS	L	2
	BC2_31518T	*MSH6* ex 1-2 genomic deletion/MLPA	posit	MSS	s	108
	BC39_33217T	*MSH6* ex 1-2 genomic deletion/MLPA	posit	MSS	ND	2
	BC40_31503T	*MSH6 c.*2983G>T (nonsense)	posit	MSS	None	5
**NC-BC**	BC24_31711T	Non-carrier	posit	MSS	N/A	1
	BC23_33198T	Non-carrier	posit	MSS	N/A	1
	BC21_31702T	Non-carrier	posit	MSS	N/A	5
	BC20_33207T	Non-carrier	posit	MSS	N/A (ds)	306
	BC19_31706T	Non-carrier	posit	MSS	N/A	2
	BC18_33215T	Non-carrier	posit	MSS	N/A	3
	BC16_33205T	Non-carrier	posit	MSS	N/A (ds)	393
	BC43_33196T	Non-carrier	posit	MSS	N/A	5
	BC15_31696T	Non-carrier	posit	MSS	N/A (ds)	161
	BC17_31692T	Non-carrier	posit	MSS	N/A	2

^*^L, LOH; s, somatic mutation; ds, double somatic mutation.

**Table 2:** Comparison of clinicopathological characteristics of patients and tumors from different groups

	LS breast carcinomas		Non-carrier breast carcinomas	LS ovarian carcinomas	LS CRC
	dMMR (*n* = 11)	pMMR (*n* = 9)	(All pMMR) (*n* = 10)	(All dMMR) (*n* = 16)	(All dMMR) (*n* = 18)
Average age of onset	53	63	59	46	44
Mean no. of non-synonymous somatic mutations	696 (174/Mb)	131 (33/Mb)	352 (88/Mb)	735 (184/Mb)	689 (172/Mb)
Proportion hypermutated [> 10 (ns) mutations per Mb]	10/11 (91%)	4/9 (44%)	3/10 (30%)	13/16 (81%)	18/18 (100%)
**Predisposing gene**					
*MLH1*	7/11 (64%)	4/9 (44%)	N/A	13/16 (81%)	12/18 (67%)
*MSH2*	2/11 (18%)	2/9 (22%)		3/16 (19%)	2/18 (11%)
*MSH6*	2/11 (22%)	3/9 (33%)		0	4/18 (22%)
**Two-hit inactivation**					
Germline mutation + LOH	6/11 (55%)	2/9 (22%)	N/A	7/16 (44%)^XXX^	9/18 (50%)^XXX^
Germline mutation + somatic point mutation (s)	4/11 (36%)	3/9 (33%)		3/16 (19%)^XXX^	6/18 (33%)^XXX^
No obvious second hit	1/11 (9%)	3/9 (33%)		1/16 (6%)^XXX^	2/18 (11%)^XXX^
ND	0	1/9 (11%)*		5/16 (31%)^XXX^	1/18 (6%)^XXX^
**Mutational signatures**					
Average 6	0,072	0,088	0,017	0,124	0,115
Average 20	0,007	0,138	0,030	0,027	0
Average 26	0,036	0	0	0,006	0,013
Average 6, 20, or 26	0,115	0,226	0,047	0,157	0,128

N/A, Not applicable, ^*^Second hit analysis was inconclusive in one tumor because LOH analysis failed, ^XXX^ Based on data in Porkka et al. 2017.

Baseline characterization included MMR status of the tumors, where the absence of MMR protein by IHC, presence of MSI, or both were required for MMR-deficiency (see Materials and Methods). While all LS-OCs and LS-CRCs were MMR-deficient (dMMR) and all NC-BCs MMR-proficient (pMMR), LS-BCs broke down into dMMR (*n* = 11) and pMMR (*n* = 9) subgroups (Table 1). LS-BCs that were dMMR were diagnosed at the mean age of 53 years vs. 63 years for pMMR LS-BCs (*p* = 0.036). NC-BCs were diagnosed at 59 years on the average (non-significant difference relative to dMMR LS-BC).

### Mechanisms of second allele inactivation of MMR genes in LS-BCs

LOH, somatic mutation, and promoter methylation were considered as possible second hits (Tables 1 and 2, Supplementary Table 1). In dMMR LS-BC, the primary mechanism of the second hit was LOH (6/11, 55%) whereas only 2/9 (22%) pMMR LS-BCs showed LOH. In pMMR LS-BC, somatic point mutation (s) of the predisposing MMR genes was the predominant mechanism (3/9, 33% vs. 4/11, 36% in dMMR LS-BC). Promoter methylation analysis by MS-MLPA indicated that none of the *MLH1*-deficient samples exhibited promoter methylation of *MLH1* as a second hit. In summary, all but one dMMR LS-BCs (10/11, 91%) had a detectable second hit, compared to 5/9 (56%) of pMMR LS-BCs (statistically non-significant). The patterns of two-hit inactivation of the predisposing MMR genes in LS-BC closely resembled those previously observed for LS-OC and LS-CRC, where a detectable second hit, mostly LOH, was present in 10/16 (63%) of LS-OCs and 15/18 (83%) of LS-CRCs (Table 2 and ref [15]).

### Mutation profiles of 578 cancer-relevant genes

#### Numbers of mutations

Somatic mutational analysis identified an average of 174 non-synonymous somatic mutations per Mb in dMMR LS-BCs, compared with 33/Mb in pMMR LS-BCs (non-significant difference) and 88/Mb in NC-BCs (borderline significant relative to dMMR LS-BC, *p* = 0.053) (Table 2). The corresponding mutation counts in LS-OCs and LS-CRCs were 184/Mb and 172/Mb, respectively.

All but one dMMR LS-BCs (10/11, 91%) were hypermutated (> 10 non-synonymous mutations per Mb). The single non-hypermutated tumor (BC11_31533T) was from a carrier of the most prevalent Finnish founder variant in *MLH1* and showed LOH at *MLH1* and absent MLH1 protein, but stable microsatellites (Table 1). The fraction of hypermutated dMMR LS-BC tumors was comparable to that of dMMR LS tumors of other organs (13/16, 81% for LS-OC and 18/18, 100% for LS-CRC) (Table 2). Among pMMR LS-BCs, 4/9 (44%) were hypermutated; of these, an inactivating somatic “hit” to the predisposing MMR gene was detectable in three and consisted of somatic mutation in all cases (Table 1).

Interestingly, the NC-BC group, too, revealed a hypermutated subset (3/10, 30%) (Tables 1 and 2). Double somatic non-synonymous point mutations in MMR genes were identified in all three hypermutated NC-BCs, affecting *MLH1* in two tumors (BC15_31696T and BC20_33207T), and *MSH2* and *MSH6* in one tumor (BC16_33205T) (Supplementary Table 2). All MMR gene mutations occurred with allele frequencies below 25% and no MSI-high or IHC abnormality was present in the tumors. It is possible that the results reflected clonal heterogeneity without actual two-allele inactivation in any subclone. To search for alternative explanations for the high mutational loads, the hypermutated pMMR breast carcinomas (LS and NC cases) were Sanger-sequenced for *POLE* exon 9 and 13 and *POLD1* exon 11; no mutations were identified in any cases successful in analyses.

#### Top mutant genes

Mutant allele frequencies of at least 25% and involvement in approximately one-third or a higher proportion of the tumors were considered as indicators of possible cancer driver nature, as outlined in our previous study [15]. In dMMR LS-BC, 18 genes fulfilled these requirements (were affected with high-frequency mutations in a minimum of 27% of the tumors; Supplementary Table 3). The 18 “top mutant” genes included 5 known to participate in epigenetic regulation and 4 involved in DNA repair, which suggested significant enrichment when compared to the shares of epigenetic regulatory genes and DNA repair genes in the entire panel of 578 genes (5/18 vs. 47/578, *p* = 0.010, and 4/18 vs. 45/578, *p* = 0.043, respectively). Details of all mutations affecting the 18 top mutant genes are described in Supplementary Table 4. Fewer top mutant genes were identified for pMMR LS-BC and NC-BC by an analogous selection procedure (Supplementary Table 3).

**Figure 1:**
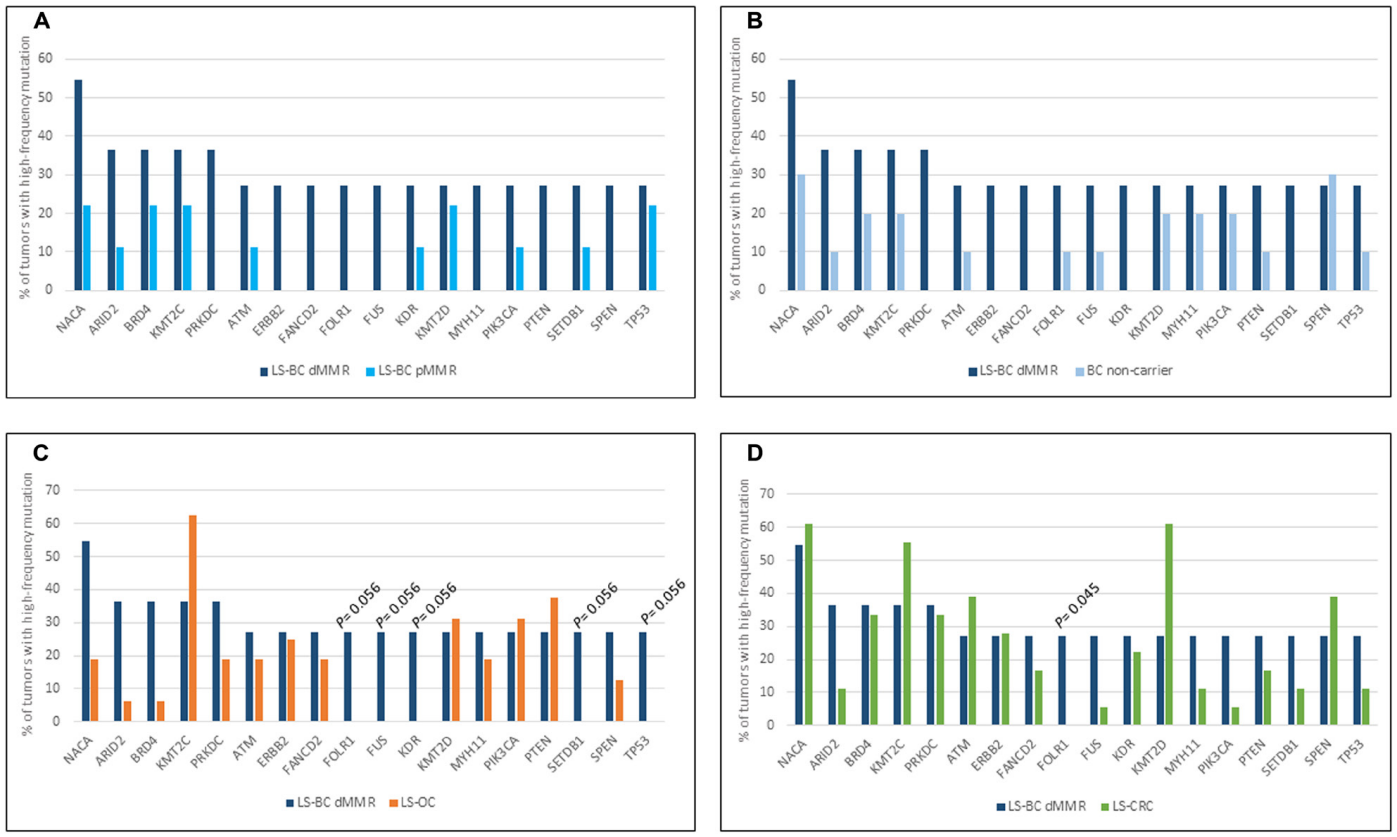
Top mutant genes across tumor types. Involvement of 18 LS-BC-associated top mutant genes (x-axis) in the dMMR subset of LS-BC (dark blue bars in (**A**–**D**), compared to the pMMR subset of LS-BC (turquoise bars in A), non-carrier BC (light blue bars in B), LS-OC (orange bars in C), and LS-CRC (green bars in D).

Figure 1 shows the involvement of the 18 dMMR LS-BC-associated genes in the remaining carcinoma groups, with each group compared against dMMR LS-BC. Due to the modest sample sizes, no high-level significant differences were observed; however, some observations can be made. MMR-proficient breast carcinoma groups (pMMR LS-BC and NC-BC) revealed comparable patterns, which often deviated from the dMMR LS-BC mutational pattern, implying the effect of MMR proficiency/deficiency (Figure 1A and 1B). The three MMR-deficient carcinoma groups (dMMR LS-BC, LS-OC, and LS-CRC) showed similarities and differences likely to at least in part reflect the tissues of origin (Figure 1C and 1D).

According to the predicted consequences, non-synonymous somatic mutations were classified into non-truncating (missense or in frame insertion/deletion) and truncating (frameshift or nonsense). As expected, the relative proportion of truncating mutations affecting our top mutant genes was higher in dMMR LS-BC compared to pMMR LS-BC (Supplementary Figure 1).

### Mutational signature analysis

**Figure 2:**
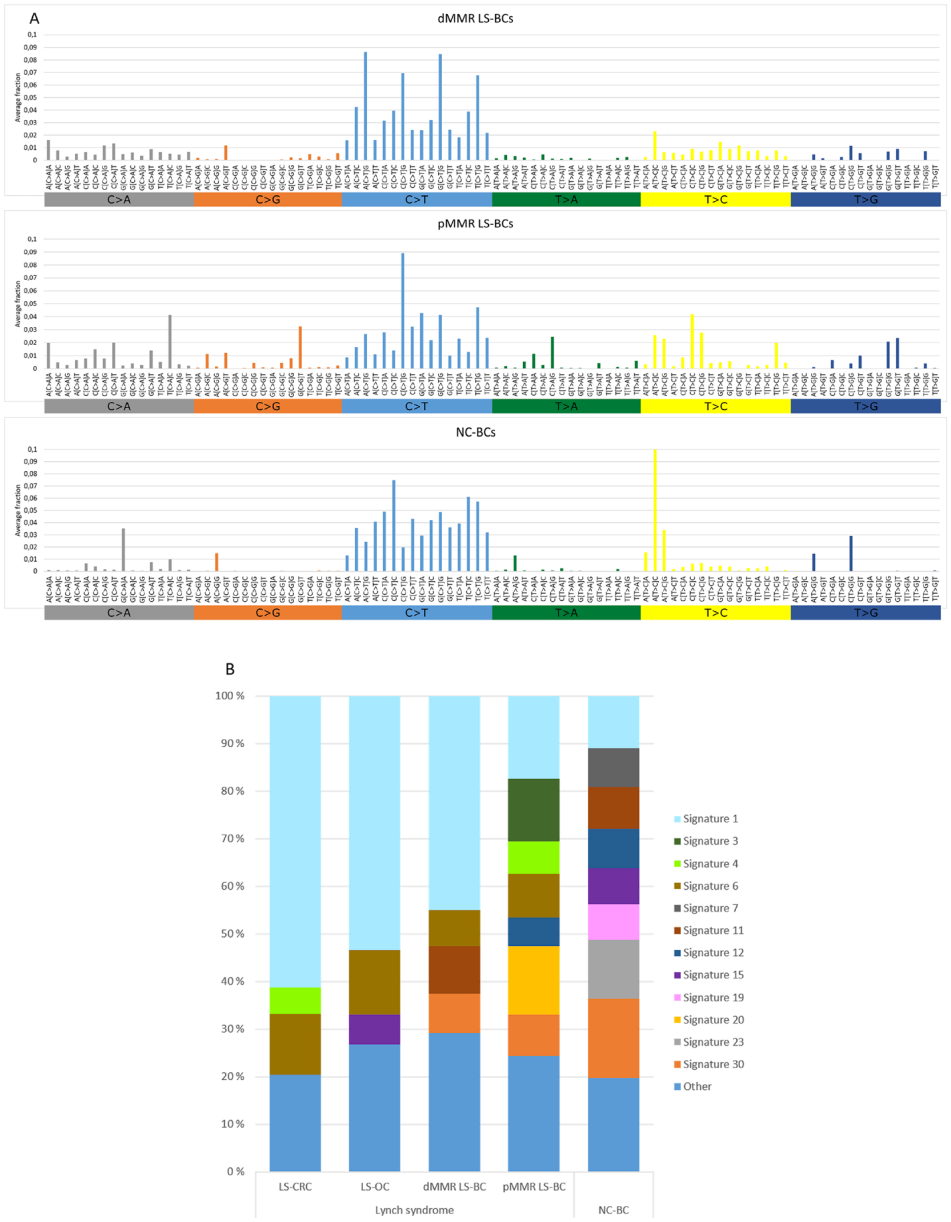
Mutational signatures of tumor groups. (**A**) Distributions of the average fractions of each of the 96 possible trinucleotide substitutions across dMMR LS-BCs, pMMR LS-BCs, and NC-BCs. (**B**) Proportions of COSMIC signatures 1–30 in LS-CRC, LS-OC, dMMR LS-BC, pMMR LS-BC, and NC-BC, based on average frequencies across each group. Signatures with average frequencies below 0.05 are combined into the ‘Other’ group.

Somatic non-synonymous (high- and low-frequency) mutations detected in the panel of 578 genes were used to determine mutational signatures for each group of tumors (Figure 2) As the total number of mutations was low in a proportion of tumors, we preferred not to compare individual tumors, but instead determined group-specific averages for mutational signatures and used these for group-wise comparisons. Figure 2A shows the distribution of the 96 possible trinucleotide mutations [16] in breast carcinomas. Previous studies have linked C > T transitions at NpCpG sites to MMR deficiency [16]. Such mutations represented the most frequent type of substitutions in both the dMMR and pMMR subsets of LS-BC and were less common in NC-BC. Thirty reference mutational signatures are indexed in COSMIC, and Figure 2B shows the relative percentages of these signatures in BC, OC, and CRC. Among the main MMR-deficiency associated signatures 6, 20, and 26 [16], signature 6 was well represented in all tumor groups from originating from carriers of inherited MMR gene defects, including LS-BC (both dMMR and pMMR), LS-OC, and LS-CRC. Signature 20 was prominent in pMMR LS-BC. When the average frequencies of signatures 6, 20, and 26 were combined, dMMR LS-BC (0.115) and pMMR LS-BC (0.226) resembled LS-OC (0.157) and LS-CRC (0.128), whereas the share of MMR-deficiency associated signatures in NC-BC was clearly lower (0.047) (Table 2). The *POLE*-associated signature 10 [16] was absent in our entire breast cancer series. Of curiosity, all breast carcinoma subgroups (LS and NC) shared a notable signature 30 (Figure 2B), recently linked to defective base excision repair characteristic of breast and other carcinomas from biallelic *NTHL1*-mutation carriers [17]. *NTHL1* was not included in our CCP panel, and the origin of signature 30 in our series remains unknown.

## DISCUSSION

The standardized incidence ratio of breast cancer in the Finnish LS families is not elevated [18]. Moreover, breast carcinoma from LS patients is not associated with any specific histological phenotype or clinical features that would distinguish it from breast carcinomas that occur sporadically. LS-associated breast carcinoma is typically ductal and diagnosed above 50 years of age on the average [19–22]. Hormone receptor status may vary: Walsh et al. [22] observed that most dMMR breast carcinomas from LS patients were hormone receptor negative compared to the pMMR subgroup, whereas the majority of LS breast carcinomas from our series were estrogen receptor-positive (15/18, 83%) and no difference between the dMMR and pMMR subgroups was observed. Like the series of Walsh et al. [22], our LS breast cancers were predominantly HER2-negative (15/18, 83%).

Microsatellite and immunohistochemical analysis divided our LS breast carcinomas into dMMR (55%) and pMMR (45%) subgroups in agreement with published studies (average 51% dMMR [6];). We have previously noted that immunohistochemical analysis is more sensitive than MSI to detect MMR deficiency in breast and other cancers from LS patients [14]. This likely reflects clonal heterogeneity characteristic of LS and sporadic MMR-deficient tumors [23, 24]. Consequently, all (100%) dMMR LS-BCs revealed immunohistochemical abnormality, but only 6 (55%) showed MSI-high. Neither MSI nor immunohistochemical change was present in the pMMR subgroup of LS-BCs.

Incorporation of mutational signatures which reflect the underlying pathophysiologic processes [16] may increase sensitivity even further. By studying large series of unselected breast carcinomas by genome-wide sequencing, Davies et al. [25] found that mutational signatures 6, 20, and 26 recognized MMR deficiency more faithfully than sequencing of MMR genes for mutations or analyzing exome data for MSI. We used non-synonymous mutations of 578 genes as the basis of mutation signature analysis. This limitation must be kept in mind when interpreting differences in mutation spectra, although targeted capture by panel sequencing and/or restriction to non-synonymous mutations have turned out informative in previous studies [26, 27]. The combined average proportion of MMR-deficiency-associated signatures, while low in NC-BC, was in our dMMR subset of LS-BC comparable to the averages observed in the established LS-spectrum tumors LS-OC and LS-CRC, and in the pMMR subset of LS-BC even higher (Table 2), suggesting that inherited MMR deficiency was likely to play an important role in the etiology of LS-BC irrespective of IHC/MSI status.

The total mutational burden offers another opportunity to reveal MMR deficiency. Nowak et al. [27] compared panel sequencing results from unselected colorectal carcinomas with immunohistochemical and MSI data and found that false negative results relative to MSI were attributable to tumor heterogeneity, whereas false positive results were explained by *POLE* mutations. Using the commonly accepted threshold of over 10 somatic mutations/Mb [28], 100% LS-CRCs and 91% dMMR LS-BCs were hypermutated, compared to 81% of LS-OCs and 44% of pMMR LS-BCs (Table 2). Importantly, the NC-BC group also had a notable hypermutated subset (3/10, 30%) in the absence of MSI or extinct MMR protein expression or evidence of DNA polymerase proofreading defects. While somatic low-frequency mutations were detected in MMR genes and might play a role, the ultimate mechanism of hypermutated phenotype in NC-BC breast carcinomas remained unsettled.

Even for such cancers whose risks have consistently been shown to be elevated in LS compared to the average population, considerable MSS fractions exist. For example, brain tumors we previously examined from LS patients all lacked MSI-high [29]. Additionally, one-fourth of colorectal adenomas developing in LS patients are pMMR by immunohistochemical and MSI analysis, suggesting that MMR deficiency is not a prerequisite for tumor formation [30, 31]. In LS, even immunoactivation may take place in premalignant lesions that have neither dMMR nor elevated somatic mutational loads [32]. Significant heterogeneity has been demonstrated on genome-wide level: genomic and transcriptomic analyses conducted by Binder et al. [33] divided LS-CRCs into two subgroups, one with high numbers of somatic mutations reminiscent of sporadic MSI CRC and another one with lower mutational loads resembling sporadic MSS CRC. As for mechanisms that might mediate predisposition to pMMR cancers in carriers of inherited MMR defects, several possibilities exist. Apart from the repair of replication errors, the MMR system has many other anti-carcinogenic functions, such as cell cycle checkpoint control in response to DNA damage [34]. For *MLH1*, the predominant predisposing gene among our LS-BC cases, it was demonstrated that even low reductions of the protein product can impair cell cycle checkpoint activation while the cells remain MMR-proficient [35]. Chromosomal segregation represents another function sensitive to the dosage of the *MLH1* gene product in an analogous manner [36]. Finally, MMR proteins participate in additional repair mechanisms, whose failure may contribute to hypermutability despite MMR proficiency; for example, MSH2 is part of BRCA1-associated genome surveillance complex that protects against DNA double-strand breaks [37]. In this context, it is of interest that the pMMR subgroup of LS-BC had a notable signature 3 (Figure 2B) which is known to be associated with defects in homologous recombination [16].

A whole-genome investigation of 560 breast carcinomas [38] identified *TP53*, *PIK3CA*, *MYC*, *CCND1*, *PTEN*, *ERBB2*, *FGFR1*, *GATA3*, *RB1*, and *MAP3K1* as the most frequently mutant genes. Of these, *TP53*, *PIK3CA*, *PTEN*, and *ERBB2* were among the top mutated genes selected by the criteria we used (at least 27% of tumors affected by mutations with allele frequency 25% or higher) (Figure 1). While seldom affected in LS-CRC and LS-OC, *TP53* was mutant in 5/20 (25%) of our LS-BCs. Interestingly, somatic *TP53* mutations in breast cancer were recently associated with immune-rich status [39]. In a function-based classification, epigenetic regulatory genes and DNA repair genes were significantly enriched as mutational targets among our LS-BC-associated genes. The histone lysine methyltransferases *KMT2C* (*MLL3*) and *KMT2D* (*MLL2*) (Figure 1) also belong to the driver genes detected by Nik-Zainal et al. [38]. Mutations in these genes may alter the expression of other genes (e. g., inactivating *KMT2C* mutations were shown to downregulate genes involved in homologous recombination-mediated DNA repair, making the tumor cells chromosomally unstable [40];) or be harmful by other mechanisms (e. g., *KMT2D* mutations were found to increase mutational burden and genome instability in cancer through transcription stress [41],). Combined with our previous findings [15], frequent mutations in epigenetic regulatory genes appear to be a common feature of LS tumors, applicable to LS-OC and LS-CRC as well.

Mismatch repair deficiency and the associated hypermutability may indicate responsiveness to PD-1 blockade, as recently reported for metastatic dMMR breast cancer [42]. Rampias T et al. [40] showed that inactivating *KMT2C* mutations (see above) caused sensitivity to PARP1/2 inhibition through synthetic lethality. Several other genes involved in LS-associated breast cancer may also be clinically actionable [43].

In conclusion, we demonstrate that LS-BCs which fell into dMMR and pMMR subsets by conventional methods shared MMR-deficiency-associated consensus signatures with the established LS spectrum tumors LS-OC and LS-CRC. Our results suggest that inherited MMR deficiency likely contributed to the development of LS-BC through disruption of MMR-related and non-MMR-related functions, thereby facilitating tumor initiation or progression. As this study was based on a modest number of cases retrieved from a national LS registry and the predisposing genes (*MLH1*, *MSH2*, and *MSH6*) were unevenly distributed, our tumors may not be considered representative of all tumors of the respective organs occurring in LS. Therefore, our results need to be confirmed in larger sample sets preferably representing multiple populations.

## MATERIALS AND METHODS

### Patients and samples

All available breast carcinomas (LS-BC) (*n* = 20) and corresponding normal DNA samples from 17 females were collected from the National LS registry of Finland (LSRFi) that includes information of approximately 300 LS families and over 1600 tested carriers of inherited MMR defects. All predisposing variants were pathogenic or likely pathogenic, representing pathogenicity classes 5 and 4, respectively. *MLH1* was affected in 9 patients (with 11 tumors) of which 7 patients had the prevalent Finnish founder variant (“mutation I”), which is a 3.5-kb genomic deletion of exon 16 and its flanking introns [44]. Four individuals (with one tumor each) had a pathogenic or likely pathogenic germline variant in *MSH2*, and one individual with two tumors had a predisposing variant in *MSH6* (Table 1). Estrogen receptor (ER) status was positive in 15 LS-BC samples, 3 tested negative and the status could not be determined for 2. Human epidermal growth factor receptor 2 (HER2) status was positive for 3, negative for 15, and could not be determined for 2 LS-BC tumors.

For comparison, 10 breast carcinomas from non-carrier members of families registered in LSRFi were included (the NC-BC group in Table 1). The ER status and HER2 status (mainly positive and negative, respectively) of NC-BCs matched with those of LS-BCs. The average age at breast cancer diagnosis was similar in LS and NC-BC groups (57 and 59 years, respectively). In addition, we analyzed 16 LS ovarian and 18 LS colorectal carcinomas (LS-OCs and LS-CRCs, respectively) from the same registry [15].

All tumor samples and the majority of normal DNA samples (19/27) were formalin-fixed paraffin embedded (FFPE) samples. DNA was extracted from selected high tumor percentage areas according to the modified protocol described by Isola *et al.* [45]. The remaining eight normal DNA samples were extracted from blood according to the non-enzymatic protocol described by Lahiri and Nurnberger [46].

This study was approved by the Institutional Review Board of the Helsinki University Central Hospital (466/E6/01). The National Supervisory Authority for Welfare and Health (Dnro 1272/04/044/07 and Dnro 10741/06.01.03.01/2015) approved the collection of archival specimens. Informed consent procedures defined by the Ethics approvals were followed in sample collection.

### MMR status of breast carcinomas

Results from immunohistochemical (IHC) analyses for MMR protein expression and microsatellite instability (MSI) analyses were available from our previous investigation [14]. Breast carcinomas were considered MMR-deficient (dMMR) when MMR protein was absent by IHC and/or the tumors showed MSI (at least one of the two mononucleotide repeat markers *BAT25* and *BAT26* was unstable).

### MLH1 promoter methylation analysis

Promoter methylation status of *MLH1* in breast cancer samples with deficient MLH1 expression was determined by methylation-specific multiplex ligation-dependent probe amplification (MS-MLPA) using SALSA MS-MLPA probemix ME001-C1 (MRC Holland, Amsterdam, Netherlands), as described in Lotsari et al. [14]. Of the two *MLH1*-associated probe pairs, the one closest to the transcription start site was considered. Methylation dosage ratio of 0.25 or higher (corresponding to at least 25% of methylated DNA) provided the best discrimination between tumor and paired normal DNA and was used as the cut-off for hypermethylation [14].

### Comprehensive cancer panel (CCP) sequencing

Tumor and matching normal DNA samples from LS-BC and NC-BC cases were sequenced in the Institute for Molecular Medicine Finland (FIMM) on Illumina HiSeq 2500 platform (San Diego, CA, USA) using Nimblegen Comprehensive cancer panel (Roche Diagnostics). The panel is a 4 Mb design covering 578 known cancer-related genes and their intronic regions compiled from the Sanger Institute Cancer Gene Census database (https://www.ncbi.nlm.nih.gov/pubmed) and the NCBI Gene tests database (https://www.ncbi.nlm.nih.gov/). ThruPLEX^®^ DNA-seq Kit was used for library preparation, and exon capture was conducted according to the manufacturer’s protocol (Rubicon Genomics). The variant calling pipeline is described in detail by Sulonen et al. [47]; in the present study, we used version 3.6. To enable comparison with breast cancer data, LS-OC and LS-CRC data generated previously [15] were re-analyzed with the VCP 3.6 pipeline. Supplementary Table 5 shows performance characteristics of breast carcinomas. The mean target coverage was 160-fold for dMMR LS-BC, 114-fold for pMMR LS-BC, and 78-fold for NC-BC. Please see ref [15]. for the performance characteristics of LS-OC and LS-CRC.

### Somatic mutation analysis of CCP data

Paired tumor and normal sample sequencing data were analyzed by the VarScan 2 mutation detection algorithm version 2.3.2 [48] to identify non-synonymous (missense, nonsense, frameshift, in-frame coding deletion/insertion and splice site) changes of somatic origin. Variants with VarScan somatic *p*-value below 0.01 were considered significant and are referred to as `somatic mutations` throughout this paper. Such variants had the possibility of being pathogenic according to traditional pathogenicity classes 3–5 and were selected for subsequent analyses. The variants were categorized based on variant allele frequency (low frequency < 0.25 vs. high frequency ≥ 0.25) and effect (truncating vs. non-truncating) as described previously [15, 49].

### POLD1 and POLE sequencing

Hypermutated pMMR breast carcinomas were screened for proofreading mutations in *POLE* exons 9 and 13 by Sanger sequencing with primers described by Church et al. [50]. *POLD1* exon 11 was examined by Sanger sequencing with primers described in Valle et al. 2014 [51].

### Two-hit inactivation of MMR genes

Somatic point mutations in MMR genes that could serve as second hits were identified from the CCP sequencing data. Loss of heterozygosity (LOH) was also evaluated as a possible second hit and the method depended on the type of the predisposing MMR gene alteration. When the predisposing MMR gene change was a point mutation, VarSeq software (GoldenHelix®) with VCP filtered CCP sequencing data (. vcf-files) was used to compare sequence data from tumor and their corresponding normal samples. The variant allele reads (Alt) to reference allele reads (Ref) ratio was determined in tumor (T) and normal (N) DNA, and LOH ratio (R) calculated as follows: R = (Alt: Ref)_T_/(Alt: Ref)_N_. The thresholds for “strict” LOH and “putative” LOH were as specified by Ollikainen et al. [52]. When the predisposing change was a large deletion, MLPA-based data (SALSA P003-C1 for *MLH1* and *MSH2* and SALSA 072-C1 for *MSH6*; MRC Holland, Amsterdam, The Netherlands) were utilized for LOH analysis and the results interpreted according to Zhang et al. [53]. “Putative” and “strict” LOH are called LOH throughout this paper. In *MLH1*-associated cases, promoter methylation of *MLH1* was tested as a second hit by MS-MLPA as described above under “*MLH1* promoter methylation analysis”.

### Definition of top-mutated genes

We used a procedure developed in our previous study [15]. In brief, for each of the 578 genes of the CCP panel, we determined the proportion of tumors having that gene in a mutant form. We focused on mutations with high (≥ 25%) variant allele frequency to increase the likelihood of clonal (driver) as opposed to subclonal (passenger) mutations [54]. Based on the distribution of the proportions of tumors with individual genes mutant, a cut-off of one-third was established to divide the genes into top-mutated and less commonly mutated categories. Finally, a pathway annotation was performed on each top gene according to GeneCards (http://www.genecards.org) and relevant publications from PubMed (https://www.ncbi.nlm.nih.gov/pubmed).

### *In silico* predictions of somatic mutations

We utilized *in silico* predictions from Varsome-database [55] to assign a pathogenic significance category for somatic variants identified for MMR genes in the second hit analysis.

### Mutational signature analysis

Mutational signatures by Alexandrov et al. [16] were determined by applying the R package deconstructSigs [56] to significant (*p* < 0.01) non-synonymous somatic mutations from VarScan2 analysis (see “Somatic mutation analysis of CCP data” above) using default parameters against signatures recognized by the COSMIC database [57]. In the analysis, deconstructSigs determines the mutational profile of tumor samples by applying a multiple linear regression model to the input data. Mutational signatures were called for each sample individually and collectively for groups of samples (BC vs. OC vs. CRC; dMMR vs. pMMR).

### Statistical analyses

Statistical analyses were performed using IBM SPSS Statistical software version 25.0 (IBM SPSS Inc., Chicago, IL, USA). The applicability of the data for parametric vs. non-parametric tests was tested first. Statistical significance of distribution of mutated genes in independent groups was evaluated using the Mann-Whitney *U* test. Pairwise comparisons of frequency data were conducted by the Fisher’s exact test. Differences with *p*-value < 0.05 (two-tailed) were considered significant.

## SUPPLEMENTARY MATERIALS













## Abbreviations

CMMRD: constitutional mismatch repair deficiency
COSMIC: Catalogue of Somatic Mutations in Cancer
dMMR: mismatch repair deficient
ER: Estrogen receptor
FFPE: formalin-fixed paraffin embedded
HER2: Human epidermal growth factor receptor 2
IHC: immunohistochemistry
LOH: loss of heterozygosity
LS: Lynch syndrome
LS-BC: Lynch syndrome breast carcinoma
LS-CRC: Lynch syndrome colorectal carcinoma
LS-OC: Lynch syndrome ovarian carcinoma
LSRFi: the National LS registry of Finland
MMR: DNA mismatch repair
MSI: microsatellite instability
MS-MLPA: methylation-specific multiplex ligation-dependent probe amplification
NC-BC: non-carrier breast carcinoma
pMMR: mismatch repair proficient
