# Supplementary material for: Does breast carcinoma belong to the Lynch syndrome tumor spectrum? – Somatic mutational profiles vs. ovarian and colorectal carcinomas
Source: Oncotarget. 2020 Apr 7;11(14):1244–56. doi: 10.18632/oncotarget.27538 (PMC7147090; doi:10.18632/oncotarget.27538)
Supplement: Supplementary file 1 [file oncotarget-11-1244-s001.pdf]

Does breast carcinoma belong to the Lynch syndrome tumor spectrum? – Somatic mutational profiles vs. ovarian and colorectal carcinomas

SUPPLEMENTARY MATERIALS

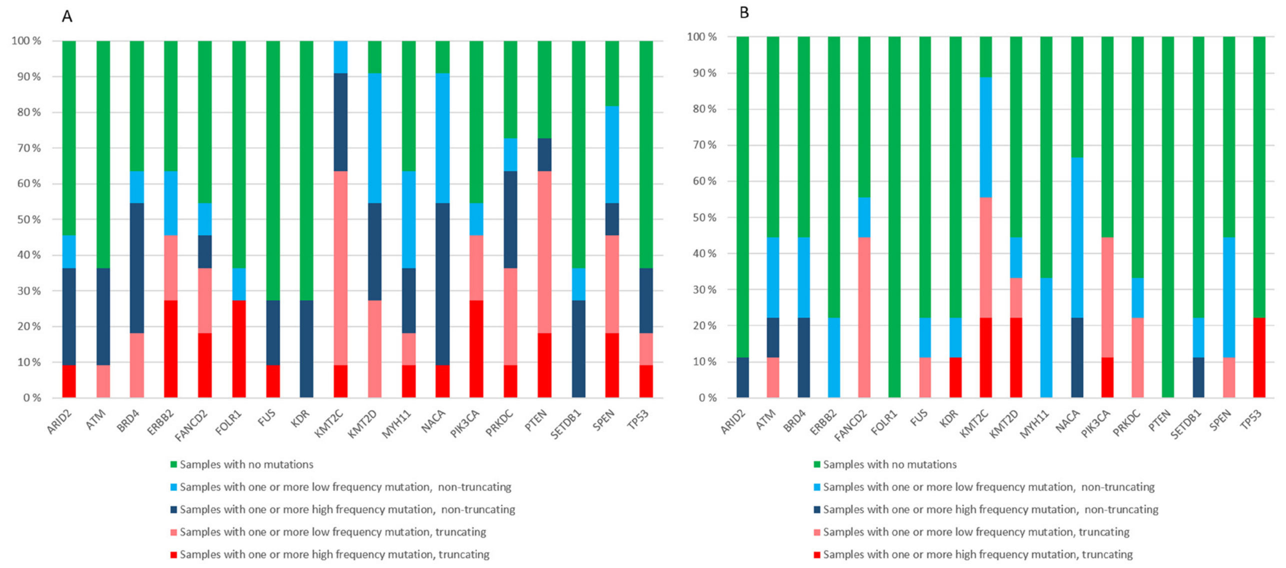

Supplementary Figure 1: Top mutated genes in LS breast cancer: truncating vs. non-truncating. Breakdown of dMMR LS-BC (**A**) and pMMR LS-BC (**B**) into five categories according to somatic mutational status of each top mutant gene specified on the x-axis. The 18 top mutant genes are those identified for dMMR LS-BC by a strategy described in Results (main paper). Somatic mutations were classified primarily by frequency (high- vs. low-frequency mutations, with variant allele frequency 0.25 as a divider) and secondarily by predicted consequence (truncating vs. non-truncating).

Supplementary Table 1: Second hit analysis of the predisposing MMR genes: somatic point mutations in LS-BCs stratified by LOH. See Supplementary Table 1

Supplementary Table 2: Somatic MMR gene mutations in breast carcinomas from proven non-carriers. See Supplementary Table 2

Supplementary Table 3: Distributions of CCP genes according to their tendency to acquire high-frequency mutations (mutant alle frequency ≥ 25%). See Supplementary Table 3

Supplementary Table 4: Specification of all somatic mutations in the top 18 genes in LS dMMR breast carcinomas. See Supplementary Table 4

Supplementary Table 5: Performance characteristics for breast carcinomas and paired normal samples. See Supplementary Table 5
